# Recent Advances in Drug Development for Alzheimer’s Disease: A Comprehensive Review

**DOI:** 10.3390/ijms26083905

**Published:** 2025-04-21

**Authors:** Haonan Xing, Song Yue, Runtian Qin, Xiaoxue Du, Yili Wu, Dongting Zhangsun, Sulan Luo

**Affiliations:** 1 Guangxi Key Laboratory of Special Biomedicine, School of Medicine, Guangxi University, Nanning 530004, China; haonanx2025@163.com (H.X.); songpolice666@163.com (S.Y.); rtianqin@163.com (R.Q.); 13156350981@163.com (X.D.); 2 Key Laboratory of Alzheimer’s Disease of Zhejiang Province, Wenzhou Key Laboratory of Basic and Translational Research for Mental Disorders, Zhejiang Provincial Clinical Research Center for Mental Health, School of Mental Health, Institute of Aging, Wenzhou Medical University, Wenzhou 325000, China; wu_yili@aliyun.com; 3 Key Laboratory of Tropical Biological Resources of Ministry of Education, Hainan University, Haikou 570228, China

**Keywords:** Alzheimer’s disease, pathogenesis, therapeutics, review

## Abstract

Alzheimer’s disease (AD) is a prevalent neurodegenerative disorder characterized by cognitive impairments such as memory loss and executive dysfunction. The primary pathological features of AD include the deposition of amyloid-beta (Aβ) plaques, the hyperphosphorylation of tau proteins leading to neurofibrillary tangles, disruptions of neuronal and synaptic functions, and chronic inflammatory responses. These multifactorial interactions drive disease progression. To date, various therapeutic agents targeting these pathological mechanisms have been developed. This article provides a comprehensive review of the pathogenesis of AD, recent advances in drug development targeting different pathways, current challenges, and future directions, aiming to offer valuable insights for clinical treatment and research.

## 1. Overview of Alzheimer’s Disease

Alzheimer’s disease (AD) is a common neurodegenerative disorder characterized by progressive cognitive decline and neuropsychiatric or behavioral changes [1,2,3]. Currently, over 55 million people worldwide are estimated to be living with AD, and this number is expected to increase significantly in the coming decades [4]. The pathological hallmarks of AD include the deposition of Aβ plaques, abnormal phosphorylation of tau proteins leading to neurofibrillary tangles, neuronal degeneration, and synaptic loss. These pathological changes contribute to the gradual decline in memory and cognitive function, ultimately impairing patients’ ability to perform daily activities and placing a significant burden on both patients and their families [5,6,7]. Current pharmacological treatments for AD, such as cholinesterase inhibitors and NMDA receptor antagonists, primarily focus on symptom management. While novel disease-modifying therapies, such as Aβ monoclonal antibodies, have shown promise, their efficacy and safety require further validation [8,9]. The primary and secondary types of AD differ fundamentally in their pathological characteristics in Table 1 [10]. Primary AD is characterized by the deposition of Aβ and neurofibrillary tangles caused by hyperphosphorylated tau proteins [11]. These lesions begin in the entorhinal cortex and gradually spread to the hippocampus and neocortex, accompanied by degeneration of cholinergic neurons and irreversible brain atrophy. In contrast, secondary AD typically lacks the typical Aβ/tau pathology, showing specific damage related to the underlying cause, such as ischemic lesions in vascular dementia or demyelination caused by metabolic disorders [12]. Its progression may be partially reversible.

## 2. Pathological Mechanisms of Alzheimer’s Disease

### 2.1. Amyloid-β Plaque Deposition

**Figure 1:**
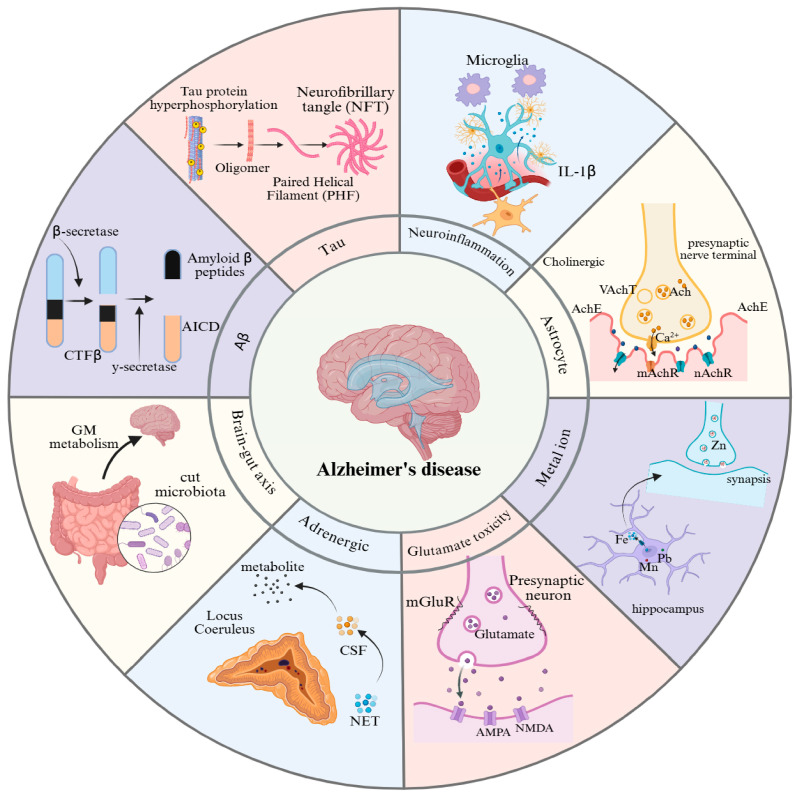
Pathogenic mechanisms of Alzheimer’s disease. This figure provides an overview of the eight major pathological features associated with AD: (1) Aβ plaque deposition, (2) abnormal tau protein hyperphosphorylation, (3) cholinergic dysfunction, (4) neuroinflammation, (5) glutamate excitotoxicity, (6) metal ion dyshomeostasis, (7) adrenergic system dysfunction, and (8) gut-brain axis dysregulation. GM: gut microbiota; NET: noradrenaline transporter; CSF: cerebrospinal fluid; NMDA: N-methyl-D-aspartic acid; AMPA: α-amino-3-hydroxy-5-methyl-4-isoxazole-propionicacid; mGluR: metabotropic glutamate; Ach: acetylcholine; AchE: acetylcholinesterase; VachT: vesicular acetylcholine transporter; mAchR: muscarinic cholinergic receptor; nAchR: nicotinic acetylcholine receptor.

The deposition of amyloid-beta plaques is one of the hallmark pathological features of AD in Figure 1 [19,20]. This process involves the abnormal production of Aβ, the formation of oligomers, their aggregation into plaques, and the subsequent downstream pathological effects. The abnormal accumulation of Aβ is a key driver of AD pathogenesis. Aβ is primarily generated through the sequential cleavage of amyloid precursor protein (APP) by β-secretase (BACE1) and γ-secretase [21,22,23,24,25]. This process yields two main forms of Aβ: Aβ40 and Aβ42. Although Aβ42 is less abundant than Aβ40, it is more neurotoxic [26]. Aβ42 has a greater propensity to aggregate, forming oligomers and fibrils that ultimately deposit as amyloid plaques [27]. Notably, Aβ oligomers, rather than fibrillar plaques, are considered the primary neurotoxic species. They directly impair neurons by disrupting synaptic function, inducing oxidative stress, and causing mitochondrial dysfunction [26]. Furthermore, Aβ deposition activates microglia and astrocytes, triggering chronic neuroinflammation that exacerbates neuronal damage [28,29].

### 2.2. Abnormal Tau Protein Hyperphosphorylation

The accumulation of amyloid-beta can induce abnormal phosphorylation of the tau protein, leading to the formation of neurofibrillary tangles (NFTs). Together with Aβ plaques, these tangles contribute to neuronal dysfunction and cell death [30]. Under normal conditions, the tau protein binds to microtubules, maintaining their stability and the structural integrity of neurons. However, in individuals with AD, tau undergoes abnormal hyperphosphorylation, which reduces its affinity for microtubules, causing microtubule disassembly and the aggregation of tau into NFTs [31,32]. The formation of NFTs disrupts the neuronal cytoskeleton, impairing intracellular transport and signal transduction and ultimately leading to neuronal apoptosis [32].

### 2.3. Cholinergic Dysfunction

The cholinergic hypothesis posits that the progression of AD is closely linked to the loss of cholinergic neurons and reduced activity of choline acetyltransferase (ChAT), leading to decreased acetylcholine (ACh) levels. According to this hypothesis, the degeneration of cholinergic neurons in the basal forebrain results in the loss of cholinergic innervation in the limbic system and neocortex, as well as impaired cholinergic neurotransmission, which contribute to cognitive decline [33]. This degeneration primarily affects memory- and cognition-related regions, such as the hippocampus and frontal cortex, leading to impaired choline uptake, reduced ACh release, receptor imbalances, and disrupted neurotrophic support [34]. The activity of cholinergic neurons in the hippocampus is closely associated with memory function. Studies have shown that damage to the septo-hippocampal cholinergic pathway significantly reduces hippocampal ACh levels, resulting in memory impairment [35]. Furthermore, restoring hippocampal ACh levels can improve memory, underscoring the critical role of hippocampal ACh in memory processes. As a key brain region involved in memory formation and consolidation, the hippocampus relies heavily on the activity of its cholinergic neurons. Additionally, dysfunction in the cholinergic system may exacerbate AD pathology by impairing synaptic plasticity and neuronal survival [36].

### 2.4. Neuroinflammation

Neuroinflammation plays a critical role in the pathological mechanisms of AD. Glial cells, the primary immune cells of the central nervous system, regulate both proinflammatory and anti-inflammatory factors [37]. In patients with AD, the deposition of Aβ plaques activates microglia, triggering the release of proinflammatory cytokines and reactive oxygen species (ROS), which exacerbate neuronal damage [38]. Additionally, astrocytes are also activated in patients with AD, releasing cytokines such as IL-1β and TNF-α, further amplifying the inflammatory response and impairing neuronal function [39]. Research indicates that neuroinflammation not only disrupts synaptic connections and impairs neural plasticity but also accelerates cognitive decline by promoting neuronal death [40]. Despite the numerous hypotheses proposed to explain the pathogenesis of AD, no definitive conclusions have been reached, likely due to the complexity of the nervous system [41]. Consequently, the high failure rate of clinical trials makes the effective validation of these hypotheses challenging.

### 2.5. Glutamate Excitotoxicity

Glutamate excitotoxicity is one of the key pathological features of AD, playing a crucial role in neuronal damage and cognitive dysfunction. Glutamate is the primary excitatory neurotransmitter in the central nervous system. At the presynaptic level, Aβ oligomers directly impair the glutamate transporter (e.g., GLT-1) in astrocytes, inhibiting glutamate reuptake and leading to an abnormal accumulation of glutamate in the synaptic cleft [42,43]. Simultaneously, presynaptic metabotropic glutamate receptors (mGluR) are activated, triggering calcium-dependent excessive release of glutamate. Postsynaptically, this excessive glutamate release causes overactivation of NMDA receptors, resulting in calcium influx and calcium overload [44]. Calcium overload further activates a series of toxic pathways, including mitochondrial dysfunction, increased ROS production, and activation of apoptosis-related proteins, ultimately leading to neuronal death [45]. Glutamate excitotoxicity not only directly damages neurons but also may contribute to the pathological progression of AD through the induction of neuroinflammation and oxidative stress [46].

### 2.6. Metal Ion Dyshomeostasis

The metal ion hypothesis emphasizes that an imbalance in the homeostasis of metal ions such as copper, iron, and zinc contributes to AD pathology through multiple mechanisms. Metal ions directly bind to Aβ, promoting its aggregation into plaques [47]. Copper and iron generate reactive oxygen species through the Fenton reaction, leading to oxidative damage. Iron and zinc activate GSK-3β kinase or interfere with synaptic function, exacerbating tau protein phosphorylation and neurofibrillary tangles [48,49]. Moreover, metal dysregulation activates microglial pro-inflammatory pathways, worsening neuroinflammation, disrupting the blood-brain barrier, and impairing synaptic function, ultimately leading to neuronal death and cognitive decline [50]. This theory highlights the pivotal role of metal ions in AD’s core pathology and lays the foundation for metal-targeted therapeutic strategies.

### 2.7. Adrenergic System Dysfunction

In Alzheimer’s disease, abnormalities in the adrenergic system are primarily manifested by early degeneration and functional disruption of the locus coeruleus [51]. Neurons in the locus coeruleus show neurofibrillary tangles and selective loss in the early stages of AD, leading to a significant reduction in the synthesis of norepinephrine (NE) in the brain, accompanied by decreased activity of tyrosine hydroxylase (TH) [52,53]. The loss of NE exacerbates neuroinflammatory responses due to its role in inhibiting the overactivation of microglial cells. The decline in NE levels may contribute to a vicious cycle of Aβ deposition and tau protein hyperphosphorylation [54]. Additionally, NE regulates synaptic plasticity and the expression of genes related to neuronal survival, and its deficiency may directly accelerate neurodegeneration in the hippocampus and prefrontal cortex [55].

### 2.8. Gut-Brain Axis Dysregulation

The pathological mechanisms of Alzheimer’s disease are closely linked to disruptions in the gut-brain axis, primarily involving dysbiosis in the gut microbiome and its mediation of metabolic, immune, and neuroinflammatory cascades [56]. In AD patients, the gut microbiome is characterized by a reduction in beneficial bacteria (e.g., Bifidobacterium, Lactobacillus) and an overgrowth of lipopolysaccharide (LPS)-producing pathogenic bacteria (e.g., Escherichia coli, Clostridia) [57,58]. This ecological imbalance affects the central nervous system through multiple pathways. These mechanisms collectively form a complex network by which the gut-brain axis mediates AD pathology, driving disease progression in a systematic manner.

## 3. AD Drug Development

**Figure 2:**
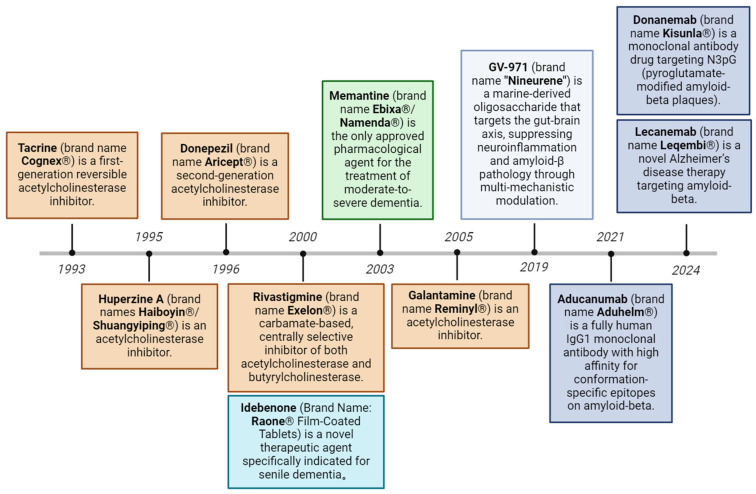
Evolution of approved therapeutic drugs for Alzheimer’s disease. Current therapeutic strategies include acetylcholinesterase inhibitors, NMDA receptor antagonists, amyloid-targeting monoclonal antibodies, reactive oxygen species modulators, and gut-brain axis-targeting modulators.

The development of therapeutic drugs for AD has been the focus of global medical research. As shown in Figure 2, 12 drugs have been approved by the U.S. Food and Drug Administration (FDA) to treat AD as of 2024. A total of 127 drugs are being investigated in 164 clinical trials for AD. Forty-eight phase III trials involving 32 drugs, 90 phase II trials involving 81 drugs, and 26 phase I trials involving 25 drugs are being conducted. In 2023–2024, the FDA approved several new drugs, including lecanemab and donanemab, marking a new phase in the treatment of AD. However, these drugs can only control or delay the development of the disease, cannot reverse or cure it, and often have certain side effects, such as gastrointestinal adverse reactions, diarrhea, nausea and vomiting and other symptoms. However, the actual clinical application of drugs is still relatively limited, and the efficacy and safety of most drugs are still continuously being verified. The investment in drug development is also considerable; the National Institutes of Health (NIH), in accordance with different research and disease types, has published funding estimate data, and Alzheimer’s disease research funding data on the website of the Alzheimer’s Association indicate that the total investment in AD drug development in the United States is approximately $6 billion per year. According to the Pharmaceutical Manufacturing and Research Association, more than $600 billion has been invested in Alzheimer’s disease research and development worldwide. However, due to the complexity of the disease, success rates remain low, with failure rates as high as 95% [59,60]. Overall, although some progress has been made in AD drug development, many challenges remain in achieving more widespread and effective clinical treatments.

### 3.1. Research and Development of AD Drugs Targeting Aβ

Recent studies [61] have shown that Aβ-targeting therapies can reduce the cerebral plaque burden and slow disease progression in AD patients. Insights from both their successes and limitations provide valuable guidance for future drug development. Table 2 summarizes the current clinical research on Aβ-targeting therapies for AD. Aβ was first isolated in 1984 by Glenner and Wong from the postmortem brains of individuals with AD and Down syndrome [62,63]. Current clinical strategies for targeting Aβ include (1) reducing Aβ production, as exemplified by agents such as lenalidomide [64]; (2) increasing the clearance of Aβ or its aggregates, as observed with bexarotene [65]; (3) disrupting or inhibiting Aβ aggregation, including compounds such as ALZ-801 [66] and PBT2 [67]; and (4) mitigating the toxic effects of Aβ aggregates, as represented by ALX-001 [68] and CT1812 [69].

To date, three Aβ-targeting drugs have been approved by the FDA for the treatment of AD. Among these, aducanumab was approved on 7 June 2021 as a human monoclonal antibody that selectively targets aggregated Aβ [70]. The Aβ-reducing activity of aducanumab likely involves multiple complex mechanisms. Activated microglia appear to surround the dense cores of processed plaques, potentially isolating them from the surrounding neuropil. Two phase 3 clinical trials, EMERGE and ENGAGE, evaluated the efficacy and safety of aducanumab in patients with early AD, involving 1638 and 1647 participants, respectively. Patients were randomized 1:1:1 to receive low-dose aducanumab (3 or 6 mg/kg target dose), high-dose aducanumab (10 mg/kg target dose), or a placebo via IV infusion every four weeks for 76 weeks. Based on changes in the Clinical Dementia Rating Scale-Sum of Boxes (CDR-SB) score from baseline to week 78, the FDA granted accelerated approval to aducanumab in 2021, despite divergent outcomes between the EMERGE and ENGAGE trials [71]. However, the FDA mandated a post approval confirmatory trial, ENVISION, to verify the clinical benefits, with results expected in 2026 [72]. In 2022, an expert panel of clinicians updated recommendations for the appropriate use of aducanumab. The panel advised strict patient selection criteria to align with the populations studied for efficacy and safety. Titration to the highest dose is recommended to maximize the therapeutic potential while closely monitoring for amyloid-related imaging abnormalities (ARIAs), which occur more frequently at higher doses. Pretreatment magnetic resonance imaging (MRI) is essential, and treatment should be interrupted or discontinued in patients who experience symptomatic moderate-to-severe ARIAs [73].

Lecanemab was approved on 9 January 2024, as a humanized IgG1 monoclonal antibody [74]. It exhibits high selectivity for soluble Aβ aggregates and moderate selectivity for fibrillar amyloid [75]. The CLARITY AD trial, an 18 month study involving 1795 participants with mild cognitive impairment (MCI) or mild dementia, evaluated the efficacy of lecanemab. Participants received weekly intravenous infusions of 10 mg/kg lecanemab, with all participants confirmed to have amyloid deposition via PET scans prior to enrollment. The trial results demonstrated significant reductions in the amyloid plaque burden compared with that in the placebo group. Cognitive decline, as measured by the CDR-SB, slowed by 27%, whereas the decline measured by the Alzheimer’s Disease Cooperative Study–Activities of Daily Living Scale for Mild Cognitive Impairment (ADCS-MCI-ADL) slowed by 37%. Additionally, cognitive decline, as assessed by the Alzheimer’s Disease Assessment Scale–Cognitive Subscale (ADAS-Cog), was reduced by 26%. These findings indicate that lecanemab treatment significantly reduces the number of amyloid plaques and slows clinical deterioration [76].

Donanemab was approved on 17 December 2024 as a monoclonal antibody designed to clear cerebral amyloid plaques [77]. It selectively targets the insoluble, modified N-terminal truncated forms of Aβ found exclusively in amyloid plaques. By binding to these truncated Aβ species, donanemab promotes plaque removal through microglia-mediated phagocytosis. The phase 3 TRAILBLAZER-ALZ trial indicated a significant clinical improvement at 76 weeks, showing efficacy in patients with early symptomatic Alzheimer’s disease and confirmed amyloid and tau pathology [31].

Although the data from some clinical studies are positive, these drugs have certain limitations, such as side effects associated with lecanemab that are similar to those associated with aducanumab. The most common side effect was infusion-related, with approximately 40% of patients requiring the use of acetaminophen or antihistamines prior to infusions. The incidence of amyloid-related image abnormality of edema or effusion (ARIA-E) or amyloid-related imaging abnormalities-hemosiderin (ARIA-H) was 17% in lecanemab-treated subjects and 9% in the placebo group, especially in those who carried the homozygous ApoE4 allele. Professors Whitehouse and Saini argued that approval of aducanumab should be withdrawn based on poor efficacy, conflicting data, and the risk of side effects [78]. Three members of the FDA Advisory Committee concluded that the drug, while approved, lacked efficacy [79]. Many different opinions on aducanumab have been reported, as phase 3 clinical trials have conflicting results [80], and A. Brandon E. Turner has raised the possibility of racial disparities in the use of drugs in clinical trials [81]. In clinical trials of lecanemab, approximately 77% of participants were white, followed by 17% Asian and only 2.6% Black [74]; thus, population differences should also be considered in the development of Aβ drugs. Overall, the efficacy of anti-Aβ drugs in the treatment of AD remains controversial. We believe that the beneficial effects of approved drugs are modest, although the data suggest that anti-Aβ agents slow the rate of functional and cognitive decline in some patients.

**Table 2:** Aβ-targeting therapeutics for Alzheimer’s disease.

Drug Name	Developing Company	Development Phase	References
AV-1959D	Institute for Molecular Medicine (Helsinki, Finland)	Phase I Clinical Trial	[82]
ALX-001	Bristol-Myers Squibb Co. (New York, NY, USA)	Phase I Clinical Trial	[68]
amyloid-beta peptide oligomer	Heinrich Heine Universitat Dusseldorf (Düsseldorf, Germany)	Phase II Clinical Trial	[83]
PBT-2	Alterity Therapeutics Ltd. (Melbourne, VIC, Australia)	Phase II Clinical Trial	[67]
UB-311	United Biomedical Inc. (Hauppauge, NY, USA)	Phase II Clinical Trial	[84]
bexarotene	Case Western Reserve University (Cleveland, OH, USA)	Phase II Clinical Trial	[65]
Lenalidomide	Celgene (Basking Ridge, NJ, USA)	Phase II Clinical Trial	[64]
sabirnetug	Acumen Pharmaceuticals Inc. (Charlottesville, VA, USA)	Phase III Clinical Trial	[85]
ALZ-801	BELLUS Health Inc. (Laval, QC, Canada)	Phase III Clinical Trial	[66]
simufilam	Cassava Sciences Inc. (Austin, TX, USA)	Phase III Clinical Trial	[86,87]
crenezumab	AC Immune SA (Lausanne, Switzerland)	Phase III Clinical Trial	[88]
lecanemab	Bioarctic AB (Stockholm, Sweden)	Market Authorization	[74]
aducanumab	Neurimmune Holding AG (Zurich, Switzerland)	Market Authorization	[70]
donanemab	Eli Lilly and Co. (Indianapolis, IN, USA)	Market Authorization	[77,89]

### 3.2. Research and Development of Tau-Targeted AD Therapeutic Drugs

The potential role of tau biomarkers in the development of tau-targeted therapeutics is indeed critical, especially in the treatment of AD. As the understanding of tau aggregation and posttranslational modification has improved, the first generation of tau-targeted interventions has focused on mechanisms associated with reversing tau aggregation, including posttranslational modifications such as hyperphosphorylation, or directly blocking tau aggregation. For example, nicotinamide has been studied as a potential intervention agent to reduce the hyperphosphorylation of the tau protein [90]. Current research is further advanced, with new clinical strategies such as tau immunotherapy and methods to target tau production. These strategies slow the progression of the disease by removing or blocking the accumulation of tau aggregates. Immunotherapy uses antibodies to directly target tau in an attempt to mitigate or stop damage to the nervous system caused by tau aggregation, whereas strategies targeting tau production focus on reducing tau production or intervening before it forms aggregates. Most tau immunotherapies currently in development target N-terminal or MTBR domains, such as ACl-35 [91], tilavonemab [92], bepranemab [93], semorinemab [94], and posdinemab [95]. In Table 3, a brief overview of current clinical studies of tau-targeted drugs for the treatment of AD is provided for comparison.

Many limitations still exist in the clinical study of the use of tau-targeted drugs for the treatment of AD. For example, the overall safety of leuco-methylthioninium (LMT) as a monotherapy has several drawbacks, with adverse events affecting the gastrointestinal and urinary tracts being the most common and the most common reason for the cessation of high doses of LMTM [96]. Similarly, zagotenemab failed to significantly slow clinical disease progression. Imaging biomarkers and plasma NfL results revealed no evidence of pharmacodynamic activity or disease modification [97]. In general, the pharmacological mechanism of tau is not clear, and clinical studies on the efficacy of most drugs in patients with a moderate tau burden are not satisfactory. The binding properties of different anti-tau molecules may prove effective in ongoing and future AD trials.

**Table 3:** Tau-targeting therapeutics for Alzheimer’s disease.

Drug Name	Developing Company	Development Phase	References
ACI-35	AC Immune SA (Ecublens, Switzerland)	Phase I Clinical Trial	[91]
PRX-005	Prothena Corp plc (Dublin, Ireland)	Phase II Clinical Trial	[98]
semorinemab	AC Immune SA (Ecublens, Switzerland)	Phase II Clinical Trial	[94]
AADvac1	Axon Neuroscience SE (Larnaca, Cyprus)	Phase II Clinical Trial	[99,100]
tilavonemab	Washington University in St. Louis (St. Louis, MO, USA)	Phase II Clinical Trial	[92]
bepranemab	UCB SA (Brussels, Belgium)	Phase II Clinical Trial	[93]
gosuranemab	Bristol-Myers Squibb Co. (New York, NY, USA)	Phase II Clinical Trial	[101,102]
zagotenemab	Eli Lilly and Co. (Indianapolis, IN, USA)	Phase II Clinical Trial	[97]
hydromethylthionine mesylate	TauRx Therapeutics Ltd. (Aberdeen, UK)	Phase III Clinical Trial	[96,103]
JNJ 63733657	Janssen Research and Development LLC (Bilse, Belgium)	Phase III Clinical Trial	[95]
IONIS BIIB4RX	Ionis Pharmaceuticals Inc. (Wilmington, DE, USA)	Phase III Clinical Trial	[104]

### 3.3. Research and Development of Therapeutic Drugs Targeting the Cholinergic System in AD Patients

The development of cholinergic system-targeting drugs for the treatment of AD has achieved certain results over the years. Cholinesterase inhibitors can increase ACh levels in the synaptic gap and partially improve cognitive symptoms in patients with AD [105]. Examples include the classical cholinesterase inhibitors tacrine [106], donepezil [107,108], galantamine [109], and rivastigmine [110,111]. Human studies evaluating the neuropathological diagnosis of Alzheimer’s disease have shown that cholinergic lesions appear as early as the asymptomatic or prodromic phase of the disease and are primarily presynaptic rather than postsynaptic. Table 4 provides a brief overview of current clinical studies of cholinergic system-targeting drugs for the treatment of AD for comparison.

Tacrine was approved by the FDA on September 9, 1993. Tacrine is a reversible anticholinesterase (AChE) inhibitor that increases the content of ACh by inhibiting AChE and can inhibit AChE in plasma and tissues. The activation of M and N receptors promotes ACh release [112]. A clinical trial investigated the efficacy and safety of high-dose tacrine for 30 weeks in patients with suspected AD [106]. A total of 663 patients were enrolled. Group 1 received the placebo, Group 2 received 40 mg/d tacrine for 6 weeks followed by 80 mg/d tacrine for 24 weeks, and Groups 3 and 4 received 40 mg/d tacrine for 6 weeks, 80 mg/d tacrine for 6 weeks, and 120 mg/d tacrine for 6 weeks. For Group 3, 120 mg/d tacrine was maintained for 18 weeks; after receiving a dose of 120 mg/d tacrine for 6 weeks, the dose administered to Group 4 was titrated to 160 mg/d tacrine for the final 12 weeks. Assessments based on the Clinician Interview Scale Universal (CIBI), ADAS-Cog, and Final Comprehensive Consensus Assessment (FCCA) showed that tacrine improved outcomes in AD patients [106].

Huperzine A was approved by the FDA on December 31, 1995, and it is an effective monomer isolated from the Chinese herb Melagna. Huperzine A has a highly selective inhibitory effect on acetylcholinesterase, thereby reducing the hydrolysis of acetylcholine and enhancing the function of choline by activating N receptors or M receptors on the presynaptic membrane and antagonizing M receptors on the postsynaptic membrane. In addition, through the supplementation of acetylcholine precursors, the synthesis of acetylcholine is increased and cholinergic neurons are excited, thus enhancing the learning and memory abilities of AD patients and improving cognitive and behavioral functions [113]. A clinical trial investigated the clinical efficacy and safety of huperzine A in the treatment of patients with mild to moderate AD. A total of 202 AD patients were randomized to receive either huperzine A or placebo for 12 weeks. One hundred patients in the huperzine A group and 102 patients in the huperzine A group were evaluated every 6 weeks after treatment with 400 µg/d. The evaluation was conducted based on the Mini-Mental State Examination (MMSE), cognitive subscale of the ADAS-COG, ADAS noncognitive subscale (ADAS-Non-COG), activities of daily living scale (ADL), and impression change scale (CIBICplus). Huperzine A can significantly improve cognitive function, behavioral and mood disorders, activities of daily living, and general function in AD patients, with a good safety profile [114].

Donepezil was approved by the FDA on 23 December 1996. Donepezil is a specific reversible central AChE inhibitor. Donepezil can inhibit the hydrolysis of acetylcholine by cholinesterase, has the strongest inhibitory effect on AChE in brain tissue, which lasts for a long time without adverse reactions in the peripheral nervous system, and can increase the expression level of acetylcholine in the synapses of the central nervous system, especially the cerebral cortex and basal ganglia [115]. The exchange of information between neurons can be improved by increasing the number of synaptic connections [116]. Clinical trials have investigated the efficacy and safety of different doses of donepezil in patients with mild to moderate AD (*n* = 154). Control patients were treated with a placebo (*n* = 162) or 10 mg/d donepezil (*n* = 157) for 24 weeks, followed by 6-week single-blind placebo clearance. The ADAS-cog, Clinician Interview-Based Assessment of Change (CIBIC plus), CDR-SB, and patient-rated quality of life (QoL) were evaluated and analyzed. The 5 mg/d donepezil group exhibited significantly improved cognitive function, as measured by the ADAS-cog, at weeks 12, 18, and 24 [117].

Rivastigmine was approved by the FDA on 14 August 2000. Rivastigmine is a carbamate-selective acetylcholinesterase inhibitor that can selectively enhance the effect of acetylcholine on the cerebral cortex and hippocampus but does not act on AChE in other brain regions, which can improve cognitive efficiency. Over time, this inhibition reverses a property that allows better control of ACh levels in the brain, thereby minimizing ACh toxicity [118]. Clinical studies have used 13.3 rivastigmine patches at a dose of 4.6 mg/24 h in patients with severe Alzheimer’s disease. Of the 716 patients randomly assigned, 356 received the 13.3 mg/24 h patch, and 360 received the 4.6 mg/24 h patch for 24 weeks [119]. Based on the analysis of the Severe Impairment Battery (SIB) and the ACTivities of daily living and cognitION (ACTION), a high dose of 13.3 mg/24 h rivastigmine patch can reduce cognitive decline in patients with severe Alzheimer’s disease dementia. A phase 3 clinical study examined the safety and tolerability of switching from donepezil to rivastigmine patches and rivastigmine capsules in patients with mild to moderate Alzheimer’s disease. The patients who received rivastigmine patches (*n* = 261) or rivastigmine capsules (*n* = 331) had mean ages of 77.3 ± 8.0 and 78.1 ± 7.8 years, respectively, and received treatment for 26 weeks [120]. Patients switched from donepezil to the cabalatin transdermal patch (4.6 mg/24 h) (immediately or 7 days after discontinuation). Safety outcomes included adverse events (AEs), AE-induced discontinuation, and severe AEs (SAEs). The analysis revealed that the rivastigmine patch seems to be better tolerated than rivastigmine capsules.

Galantamine was approved by the FDA on 1 April 2005. Galantamine was originally isolated from bulbs of the plants snowdrop and daffodil [121]. Clinical reports have documented the efficacy and tolerability of galantamine administered at 18, 24, and 36 mg/day for 3 months in 285 patients with mild to moderate suspected AD. Assessments based on the ADAS-cog, Clinical General Impression Change (CGIC), and PSMS (PDS) concluded that galantamine significantly improved the core symptoms of Alzheimer’s disease compared with the placebo [122]. Another study of community-dwelling patients explored the long-term efficacy of galantamine in patients with mild Alzheimer’s disease. The participants included patients with mild AD who had been treated with galantamine for up to 36 months (Alzheimer’s Disease and Related Disorders Association NINCDS-ADRDA standard). Based on changes in the ADAS-cog/11, the Bayer-ADL scale (self- and caregiver ratings), the 10-item neuropsychiatric questionnaire NPI, and the General Clinical Efficacy Rating Scale (CGI) scores and safety and tolerability measures, the researchers concluded that galantamine was generally safe and well tolerated during the three-year observation period. Improvements in cognition, behavior, and activities of daily living were observed during the 12 month treatment period [123].

Several limitations exist in the clinical study of targeted cholinergic drugs for the treatment of AD. The poor use of cholinesterase inhibitors is a very common limitation. According to a US survey of 25,561 Alzheimer’s patients, approximately 46.5% were prescribed one or more cholinesterase inhibitors. Donepezil was the most commonly used drug (68.0%), followed by rivastigmine (26.0%) [124]. Interviews and contacts with 803 physicians revealed that physicians with strict efficacy requirements for clinically relevant efficacy measures were less likely to prescribe cholinesterase inhibitors to treat AD [125]. Limitations also exist with approved drugs, such as tacrine, for which the most common side effects are gastrointestinal symptoms, elevated aminotransferase levels, and headaches [126]. For example, the results of a clinical trial of physostigmine revealed a significant increase in gastrointestinal side effects (including nausea, vomiting, diarrhea, anorexia, indigestion, and abdominal pain) in patients taking any dose of physostigmine, leading to a high dropout rate [127]. Therefore, a lower starting dose and flexible treatment plan should be considered, which may reduce the probability of adverse events. Although debate is ongoing among physicians about the efficacy of cholinesterase inhibitors in treating Alzheimer’s disease [128], most research supports the benefits of promoting cholinergic activity in Alzheimer’s disease through the use of cholinesterase inhibitors. With significant developments in treatment strategies for Alzheimer’s disease, targeted cholinergic interventions are likely to maintain their critical role in treatment.

**Table 4:** Cholinergic-targeting therapeutics for Alzheimer’s disease.

Drug Name	Developing Company	Development Phase	References
ladostigil	Hebrew University of Jerusalem (Jerusalem, Israel)	Phase II Clinical Trial	[129]
metrifonate	Bayer AG (Bayer Leverkusen, Germany)	Apply for Marketing Approval	[130]
physostigmine	Forest Laboratories Inc. (New York, NY, USA)	Apply for Marketing Approval	[127]
Donepezil	Eisai Co., Ltd. (Tokyo, Japan)	Market Authorization	[108,131]
galantamine	Janssen-Cilag Ltd. (Titesville, NJ, USA)	Market Authorization	[109]
rivastigmine	Novartis AG (Basel, Switzerland)	Market Authorization	[111,132,133]
huperzine A	Shanghai Institute of Materia Medica of the Chinese Academy of Sciences (Shanghai, China)	Market Authorization	[114,134]
tacrine	Warner-Lambert Co. (Morris Plains, NJ, USA)	Market Authorization	[106]

### 3.4. Research and Development of AD Therapeutic Drugs Targeting Neuroinflammation

Recent findings support the role of inflammation in the pathogenesis of AD. Table 5 provides a brief overview of current clinical studies of drugs that target neuroinflammation for the treatment of AD for comparison.

The drug idebenone was approved by the FDA on 15 December 2000, based on a clinical study of two doses of 30 mg t.i.d. in patients with Dementia of Alzheimer Type (DAT) dementia or 90 mg t.i.d. of idebenone, a diagnosis based on the DSM-III-R (primary degenerative dementia) and NINCDS-ADRDA criteria (possible Alzheimer’s disease), and treatment for 6 months. The results showed that 90 mg of idebenone t.i.d. significantly improved the main efficacy variables, namely, the ADAS-Total and ADAS-Cog scores. The efficacy and safety of idebenone in the treatment of patients with DAT have been reported [135]. With respect to the question of sustained efficacy, patients with DAT dementia were administered 90 mg of idebenone t.i.d. for 24 months, and during the placebo control period (the first year of treatment), idebenone significantly improved the primary efficacy variable, ADAS-Total score, and all secondary efficacy variables in a dose-dependent manner. Further improvements were observed in most efficacy variables in the second year compared with the first year. These findings suggest that idebenone exerts its beneficial therapeutic effect on the course of the disease by slowing its progression [136].

Masitinib is an oral tyrosine kinase inhibitor that has neuroprotective effects on neurodegenerative diseases by inhibiting mast cell and microglial/macrophage activity [137]. A phase 3 clinical study discussed masitinib as adjunct therapy for patients with mild to moderate AD [138]. Patients (2:1) were randomly assigned to receive masitinib or the placebo at an initial dose of 4.5 mg/kg/day for 12 weeks and then titrated to 6.0 mg/kg/day. Assessments based on the ADAS-cog or the Alzheimer’s Disease Scale of Daily Living Cooperative Research Activity Scale (ADCS-ADL) concluded that masitinib (4.5 mg/kg/day) may be beneficial for patients with mild to moderate AD [138].

Semaglutide, a glucagon-like peptide-1 receptor agonist (GLP-1RA), is clinically utilized for type 2 diabetes and obesity management. Its safety profile in these indications is extensively documented, with gastrointestinal disturbances representing the predominant adverse effects [139]. GLP-1RAs exert systemic receptor activation, including within the central nervous system, modulating biological pathways relevant to AD pathophysiology. Mechanistically, these agents enhance glial cell homeostasis, regulate adaptive immune responses (e.g., natural killer and regulatory T cells), preserve synaptic function, and exert neuroprotective actions [140]. Notably, a Phase III trial evaluating semaglutide in early symptomatic AD demonstrated a significant reduction in dementia incidence and attenuation of neuroinflammatory biomarkers.

One of the well-known phenomena in AD pathology is that chronic local inflammatory responses occur in pathologically vulnerable areas of the brain in patients with AD. For example, the accumulation of microglia at the site of Aβ deposition exacerbates the damage caused by other causative factors of AD. Therefore, anti-inflammatory therapy should help delay the onset of AD or slow the progression of AD. This hypothesis has been tested both directly and indirectly and is generally favorable [141]. In fact, many limitations and uncertainties exist in targeting neuroinflammation to treat AD. Compared with traditional drug intervention trials, which have shown significant efficacy at 6 months to 1 year, AD-related inflammation is chronic but weak and results in significant damage over many years; thus, the duration of anti-inflammatory interventions still needs further study.

**Table 5:** Neuroinflammation-targeting therapeutics for Alzheimer’s disease.

Drug Name	Developing Company	Development Phase	References
dazucorilant	Corcept Therapeutics Inc. (Dover, DE, USA)	Phase I Clinical Trial	[142]
liraglutide	The General Hospital Corp (d/b/a Massachusetts General Hospital) (Boston, MA, USA)	Phase II Clinical Trial	[143,144]
etanercept	Immunex Corp (Thousand Oaks, CA, USA)	Phase II Clinical Trial	[145]
Curcumin	The University of Texas MD Anderson Cancer Center (Houston, TX, USA)	Phase II Clinical Trial	[146]
bezisterim	Harbor Therapeutics Inc. (Appleton, WI, USA)	Phase III Clinical Trial	[147,148]
masitinib	AB science (Paris, France)	Phase III Clinical Trial	[138]
quetiapine IR	AstraZeneca plc (Cambridge, UK.)	Phase III Clinical Trial	[149]
NE-3107	Harbor Therapeutics Inc. (San Diego, CA, USA)	Phase III Clinical Trial	[148]
semaglutide	Novo Nordisk A/S (Copenhagen, Denmark)	Phase III Clinical Trial	[150]
idebenone	Takeda Pharmaceutical Co., Ltd. (Osaka City, Japan)	Market Authorization	[135,151]

### 3.5. Research and Development of AD Drugs Targeting Glutamate Receptors

At present, the research and development of drugs targeting glutamate receptors for AD mainly focus on regulating the activity of NMDA receptors and metabolic glutamate receptors (mGluRs) to reduce the excitotoxicity of glutamate and the degree of resulting neuronal damage. Moreover, pathological changes are accompanied by changes in cations (Na^+^, K^+^, and Ca^2+^), among which the calcium permeability of NMDA receptors is the highest, and the apoptosis of nerve cells is caused mainly by calcium overload induced by the influx of large amounts of calcium ions into NMDA receptors after overactivation by glutamate. For example, the NMDA antagonist memantine hydrochloride [152] can greatly reduce the toxic effects of glutamate on peripheral nerve cells. Table 6 provides a brief overview of current clinical studies of drugs that target glutamate receptors for the treatment of AD for comparison.

Memantine was approved by the FDA on 16 October 2003. Memantine is an NMDA antagonist. The drug was able to reduce excitotoxicity in AD patients [153]. Clinical studies have reported the efficacy and safety of memantine versus the placebo in patients with mild-to-moderate AD. Patients with moderate-to-severe Alzheimer’s disease were randomly assigned to receive a placebo or 20 milligrams of memantine per day for 28 weeks. Based on clinician interview-based change impression plus caregiver input (CIBIC-Plus) and the Alzheimer’s Disease Daily Living Scale Modified for Severe Dementia Collaborative Study Activity (ADCS-AD Lsev) analysis, memantine was not associated with a significant frequency of adverse events [154].

The research and development of AD drugs that target glutamate receptors face significant limitations, and their efficacy and mechanistic complexity are particularly prominent. Based on FDA approval data, memantine, the only approved NMDA receptor antagonist, is widely used to treat patients with moderate-to-severe AD (accounting for approximately 90% of targeted glutamate therapy prescriptions), but its efficacy delays cognitive decline for only approximately 6 months and is not effective in patients with mild AD [155]. In a phase II trial of drugs such as the presynaptic glutamate release inhibitor riluzole (NCT01703117), cognitive improvement did not reach the end point despite reduced glutamate levels in the cerebrospinal fluid. The researchers suggested the use of low-dose combination therapies (such as memantine in combination with Aβ monoclonal antibodies) or the development of subunit-selective NMDA receptor modulators (such as GLUN2b-targeting drugs) to reduce off-target effects and overcome this bottleneck. Although the single intervention strategy targeting glutamate receptors is controversial, its mechanism of blocking the excitotoxic cascade is still regarded as the core link of the AD multitarget therapy system, which needs to be combined with pathological staging and biomarkers to guide precision medicine in the future.

**Table 6:** Glutamate receptor-targeting therapeutics for Alzheimer’s disease (AD).

Drug Name	Developing Company	Development Phase	References
BMS984923	Allyx Therapeutics, Inc. (New Haven, CT, USA)	Phase I Clinical Trial	[156]
Varoglutamstat (PQ912)	Vivoryon Therapeutics NV (Halle, Germany)	Phase II Clinical Trial	[157]
Memantine	Merz Pharma GmbH and Co. KGaA (Frankfurt, Germany)	Market Authorization	[158]

### 3.6. Research and Development of AD Therapeutic Drugs Targeting the Metal Ion Dyshomeostasis

Research on AD drugs targeting metal ion homeostasis has revealed the critical role of metal regulation in the disease process. Metal chelators selectively bind excess copper, iron, and zinc ions to inhibit the metal-dependent aggregation of β-amyloid and reduce oxidative stress. Examples include the copper/zinc chelators Clioquinol [159] and PBT2 [67]. Table 7 summarizes the current clinical research progress on AD drugs targeting metal ions.

The first clinical trial using iron chelators for AD treatment was conducted in 1991, employing the drug desferrioxamine [160]. Desferrioxamine is an iron chelator produced by Streptomyces, with high affinity for both trivalent iron (Fe^3+^) and aluminum (Al^3+^). A two-year, single-blind study randomly assigned 48 suspected AD patients to receive intramuscular desferrioxamine (125 mg, twice daily, 5 days per week), oral placebo, or no treatment [160,161]. Baseline cognitive measures showed no intergroup differences. Follow-up results revealed that the desferrioxamine group experienced a significantly lower rate of decline in daily living skills compared to the placebo/no-treatment group (mean *p* = 0.03, variance *p* < 0.04), with the placebo group declining at twice the rate of the desferrioxamine group. The only reported side effects were reduced appetite (4 cases) and weight loss (1 case) [160]. The study suggests that prolonged desferrioxamine treatment may delay AD progression.

PBT2 is a metal-protein attenuating compound (MPAC) that inhibits the copper (Cu^2+^) and zinc (Zn^2+^) ion-mediated toxic oligomerization of β-amyloid in Alzheimer’s disease. A phase II, double-blind, randomized trial (NCT00471211) evaluated the safety and efficacy of the metal-protein attenuating compound PBT2 in early Alzheimer’s disease patients [67]. A total of 78 patients (MMSE 20-26/ADAS-cog 10-25) were randomly assigned to the placebo, PBT2 50 mg, or PBT2 250 mg groups for 12 weeks of treatment. The results showed that the 250 mg group had a significant reduction in cerebrospinal fluid Aβ42 levels (baseline difference −56.0 pg/mL, *p* = 0.006), with a dose-dependent effect (*p* = 0.023). However, plasma AD biomarkers and metal ion levels remained unchanged. Neuropsychological tests indicated significant improvements in executive function in the 250 mg group: category fluency increased by 2.8 words (*p* = 0.041), and the time for part B of the trail-making test decreased by 48 s (*p* = 0.009). The treatment was well-tolerated, with similar rates of treatment-related adverse events across groups (48–62%) and no serious adverse events. The study suggests that PBT2 may improve AD pathology and cognitive function by modulating Aβ toxic oligomerization, providing clinical evidence for metal-targeted therapeutic strategies in AD [162].

Clioquinol is an MPAC that inhibits the binding of zinc/copper ions to Aβ, promoting its dissolution and reducing neurotoxicity. A phase II clinical trial evaluated the potential of clioquinol in treating moderate to severe Alzheimer’s disease [159]. The study enrolled 36 AD patients and randomized them into groups. In a subgroup with more severe baseline cognitive impairment (ADAS-cog ≥ 25), the clioquinol group showed significantly slower cognitive decline compared to the placebo group. Biomarkers showed that in the clioquinol group, plasma Aβ42 levels decreased, and plasma zinc levels increased, suggesting that the drug may regulate Aβ metabolism through metal ion chelation. The drug was well-tolerated, with no serious adverse reactions reported [159]. Despite the limited sample size, the study provides preliminary evidence for MPAC-targeted therapy in AD, supporting the need for large-scale clinical trials to validate its potential in improving AD pathology through regulation of metal-Aβ interactions.

The development of AD drugs targeting metal ions faces significant limitations, with controversies regarding their efficacy and the complexity of their mechanisms. The main reason is that metal ions have dual roles in AD pathology. For example, copper both promotes Aβ deposition and participates in antioxidant enzyme activity, so excessive chelation may disrupt physiological homeostasis. In late-stage AD, Aβ plaques are widely deposited, and single-target metal ion treatments are insufficient to reverse structural damage. These limitations suggest the need for more selective mental regulation strategies, combined with multi-target interventions.

**Table 7:** Metal ion-targeting therapeutics for Alzheimer’s disease.

Drug Name	Developing Company	Development Phase	References
Deferiprone	Apotex, Inc. (Toronto, Canada)	Phase II Clinical Trial	[161]
PBT2	Alterity Therapeutics Ltd. (Melbourne, VIC, Australia)	Phase II Clinical Trial	[67,162]
clioquinol	Tianjin Tianyao Pharmaceuticals Co., Ltd. (Tianjin, China)	Phase II Clinical Trial	[159]

### 3.7. Research and Development of AD Therapeutic Drugs Targeting the Adrenergic System Dysfunction

Research on drugs targeting the adrenergic system in Alzheimer’s disease has revealed the central role of norepinephrine (NE) signaling disruption in the disease pathology. Drugs targeting the NE system mainly work by supplementing NE levels, regulating receptor activity, or protecting locus coeruleus neurons, inhibiting Aβ/tau pathology, and improving cognitive and neuropsychiatric symptoms. Table 8 summarizes the clinical research progress of AD therapeutic drugs targeting the adrenergic system.

Methylphenidate (MPH) works primarily by inhibiting the dopamine transporter (DAT) and norepinephrine transporter (NET) [163]. It reduces the reuptake of dopamine (DA) and norepinephrine at the presynaptic membrane, significantly increasing the concentration of these two monoamine neurotransmitters in the synaptic cleft. Research has explored the effects of MPH on attention and emotional apathy in Alzheimer’s disease patients [163]. In a 6-week randomized, double-blind trial, 60 patients with mild to moderate AD and emotional apathy (NPI apathy ≥ 4) were enrolled and treated with either MPH (10 mg twice a day) or a placebo. Attention was assessed using the Wechsler Digit Span test (DS), and emotional apathy was measured using the Apathy Evaluation Scale (AES). The results showed that the MPH group significantly improved both the forward score and the total score on the DS compared to the placebo group. The findings indicate that MPH can independently improve attention and emotional apathy in AD patients, suggesting that these effects may be mediated by distinct neural mechanisms, providing new evidence for AD symptom management.

Atomoxetine, a norepinephrine transporter inhibitor, works by blocking the reuptake of NE at the presynaptic membrane [164]. By blocking the reuptake of NE at the presynaptic membrane, it significantly increases the concentration of NE in the synaptic cleft of the central nervous system, thereby enhancing NE-mediated signaling. Research has evaluated its potential disease-modifying effects in prodromal AD. A 12 month double-blind crossover trial was completed with 39 patients with mild cognitive impairment and AD biomarker positivity [164]. Atomoxetine significantly increased plasma and CSF norepinephrine levels and decreased CSF tau/ptau181 (*p* < 0.05) but had no effect on amyloid-β42 levels. Neuroimaging showed enhanced insular-temporal lobe connectivity and increased glucose metabolism in key temporal regions (hippocampus, fusiform gyrus, etc.), with effects lasting up to 6 months after treatment. Safety was good, with dropout rates comparable to the placebo group. This demonstrates potential disease-modifying effects in AD, but further research is needed to validate its long-term effects in slowing disease progression.

Nicergoline, an α-1 adrenergic receptor antagonist, has been studied for its effects on AD [165]. It works by antagonizing the α-1 adrenergic receptor subtype (α1-AR) and has multiple target neuroregulatory properties. A study assessed the 6 month efficacy and safety of nicergoline (30 mg twice a day) in treating mild to moderate Alzheimer’s disease [166]. In a randomized, double-blind trial, the nicergoline group showed significantly better ADAS-cog scores compared to the placebo, with notable cognitive improvement, although there were no differences between groups on the CGIC, Instrumental Activities of Daily Living (IADL), and PSMS. Secondary endpoints showed a lower incidence of non-cognitive symptoms in the nicergoline group. Completion rates were comparable between the two groups, and the incidence of adverse events was similar, with no differences in discontinuation rates. The nicergoline group experienced more metabolic-related adverse events (such as hyperuricemia), while the placebo group had more psychiatric events. The results suggest that nicergoline is well-tolerated and can specifically improve cognitive function, but larger-scale studies are needed to confirm its clinical value [166].

Increasing evidence from research indicates that drugs targeting the adrenergic system are gaining recognition for AD treatment. However, some AD drugs targeting the adrenergic system have failed in clinical trials. For example, α2-adrenergic receptor agonists (such as guanfacine) have shown cognitive improvements in animal models, but in AD patients, their lack of target specificity and suppression of NE release from the locus coeruleus counteract the therapeutic effect, alongside serious side effects such as hypotension and sedation [167]. These failure cases highlight core issues such as misunderstandings of mechanisms, the complexity of receptor subtype functions, and improper intervention timing.

**Table 8:** Adrenergic system dysfunction therapeutics for Alzheimer’s disease.

Drug Name	Developing Company	Development Phase	References
Methylphenidate	Novartis Pharma AG (Basel, Switzerland)	Phase II Clinical Trial	[163]
atomoxetine	Eli Lilly and Co. (Indianapolis, IN, USA)	Phase II Clinical Trial	[164]
Nicergoline	Ildong Pharmaceutical Co., Ltd. (Seoul, Republic of Korea)	Phase II Clinical Trial	[168]

### 3.8. Research and Development of AD Therapeutic Drugs Targeting the Gut-Brain Axis

Recent advances in AD research have identified pharmacological approaches modulating the microbiota-gut-brain axis as a transformative frontier. The pathophysiological foundation centers on gut microbial regulation of cerebral inflammatory cascades and Aβ aggregation through tripartite mechanisms: (i) microbial metabolites (e.g., bile acids, trimethylamine N-oxide) influencing glial phagocytic activity and Aβ clearance [169]; (ii) immune mediators (IL-6, IFN-γ) propagating neuroinflammation via circulatory pathways [170]; and (iii) enteroendocrine signaling molecules (e.g., serotonin, ghrelin) modulating hippocampal neuroplasticity through vagal afferents [171]. Marine-derived bioactive compounds, with their inherent structural diversity and polypharmacological properties, present unparalleled opportunities for multitargeted intervention across these interconnected pathways.

GV-971, a marine-derived oligosaccharide, was approved on 2 November 2019. It is specifically formulated as a mixture of linear acidic oligosaccharides [172]. Its innovative mechanism involves gut microbiota remodeling through selective suppression of pathogenic bacterial metabolites (phenylalanine/isoleucine), which attenuates peripheral Th1 lymphocyte infiltration into the central nervous system (CNS) and subsequent activation of microglial NLRP3 inflammasomes [173]. Preclinical validation in transgenic AD murine models demonstrated dose-dependent reductions in both Aβ plaque burden (40% vs. controls) and p-tau levels (Ser396/404 phosphorylation sites), concomitant with Morris water maze performance enhancement [174]. Confirmatory Phase III trial data from a 36-week, double-blind study revealed clinically meaningful differences in ADAS-cog12 scores between GV-971 (900 mg/day) and placebo cohorts, with sustained cognitive benefits and ≤3% incidence of gastrointestinal adverse events [175]. This therapeutic paradigm underscores the capacity of marine-origin molecules to simultaneously engage microbial ecology and neuroimmunological pathways, transcending conventional single-target amyloidocentric approaches.

Despite the approval of FDA-approved drugs, there is still no curative treatment available. Probiotic formulations have not significantly improved cognitive function in AD patients in clinical trials [176]. This may be due to unclear mechanisms of the gut-brain axis, inter-individual microbiome differences leading to unstable efficacy, and insufficient regulation of key metabolites, which are unable to effectively cross the blood-brain barrier or inhibit neuroinflammation. These limitations reflect the complexity of multi-target and cross-system regulation in the gut-brain axis, necessitating the integration of multi-omics precision stratification and early intervention strategies.

## 4. Challenges in AD Drug Development

Although the development of AD drugs has continued to advance, the number of drugs that can successfully enter clinical application and achieve significant efficacy is very limited. At present, AD drug research and development faces the following main problems: 1. The pathogenesis of this disease is complex and diverse and includes the Aβ hypothesis, tau hypothesis, cholinergic hypothesis, vascular hypothesis, and inflammation hypothesis. Therefore, it cannot be completely cured, and most drugs achieve remission only [177]. The unclear pathogenesis directly leads to a lack of clear targets for drug development, and many drugs fail to achieve the expected effect in clinical trials [178]. 2. The genetic and environmental heterogeneity of disease are notable issues. The vast majority of cases are sporadic AD. Sporadic AD is a complex multigene disease, and its onset is jointly influenced by many factors, such as heredity, the environment, and lifestyle [179,180]. This heterogeneity makes the etiology of AD more complex and difficult to treat with a single target or single mechanism. In addition, the genetic background and environmental exposure of different patients vary greatly, resulting in different drug efficacies and further increasing the difficulty of drug development. 3. Significant clinical heterogeneity has been observed. The clinical manifestations of AD are highly heterogeneous. In addition to typical memory disorders, some patients may also present a variety of symptoms, such as visual-spatial disturbances, psychobehavioral symptoms (such as depression, anxiety, and hallucinations), aphasia, and apraxia [181]. This phenomenon of homogeneity and heterogeneity makes the diagnosis and treatment of AD more complicated. Drugs that target a single symptom may not improve a patient’s overall condition, and the development of multitarget drugs faces enormous technical and clinical challenges. 4. Comorbidities have been documented. AD patients often have other diseases, especially cerebrovascular diseases and other neurodegenerative diseases (such as Parkinson’s disease, Lewy body dementia, etc.). These comorbidities complicate the treatment of AD. For example, cerebrovascular disease may aggravate cognitive dysfunction in AD patients, and the pathological process of AD may also affect cerebrovascular function [182,183]. Therefore, when treating AD, the overall health status of patients must be considered, which places greater requirements on the safety and effectiveness of drugs. 5. Problems associated with drug delivery and the blood-brain barrier have been identified. AD is a disease of the central nervous system, and drugs need to cross the blood-brain barrier (BBB) to work [184]. However, the presence of the BBB greatly limits the efficiency of drug delivery within the brain. Many drugs that have shown potential in vitro or in animal models have failed to translate into clinical use due to their inability to penetrate the BBB effectively. 6. Reliable biomarkers are lacking. The early diagnosis of AD and assessment of drug efficacy depend on reliable biomarkers [185]. Despite recent advances in cerebrospinal fluid detection and imaging techniques for Aβ and tau proteins, biomarkers that can fully reflect the pathological process and disease progression of AD are still lacking [186]. Thus, an accurate evaluation of the efficacy of drugs during drug development is difficult, limiting the implementation of early intervention strategies.

Although FDA-approved drugs are currently on the market, radical treatment is still lacking. Treatments for AD focus on relieving symptoms (such as improving cognitive function with cholinesterase inhibitors) or slowing disease progression (such as drugs targeting Aβ or tau proteins). However, none of these methods can fundamentally cure the disease. Radical treatments need to address the source of the disease, such as repairing disease-causing gene mutations through gene-editing technology or repairing damaged neurons through nerve regeneration technology. However, these technologies are still in the research stage and are still a long way from clinical application.

## 5. Prospects for AD Drug Research and Development

### 5.1. Precision Medicine and Biomarkers

The rise of precision medicine provides a new direction for the treatment of AD, the core of which is to achieve disease typing and early intervention through multiomics data integration and biomarker technology. Multiomics analyses based on artificial intelligence (AI), such as genomic, proteomic, metabolomic, and neuroimaging data, can identify the molecular subtypes of AD, such as inflammatory dominance, synaptic dysfunction, and metabolic abnormalities, laying the foundation for personalized targeted therapies [187]. For example, a study examining the cerebrospinal fluid proteome revealed that elevated levels of specific inflammatory factors (e.g., IL-6 and TNF-α) were associated with rapidly progressive AD, suggesting that anti-inflammatory therapy may be effective for this subtype. Moreover, noninvasive biomarker detection techniques have developed rapidly, and the detection sensitivity of phosphorylated tau (p-tau217) and the Aβ42/40 ratio in blood has approached that of a cerebrospinal fluid analysis [188]. In the future, combined with AI-driven dynamic monitoring platforms, a paradigm shift from “postdiagnostic treatment” to “predictive intervention” is expected.

### 5.2. Novel Treatment Strategies

For the complex pathological network of AD, novel treatment strategies focus on multitarget collaborative interventions and delivery technology innovation. By simultaneously acting on Aβ, tau, and neuroinflammatory pathways, multitarget drug design could disrupt the pathological cascade. For example, combination therapy with the anti-Aβ monoclonal antibody donanemab and the anti-tau oligomer E2814 (NCT05026866) had a synergistic effect in a phase II trial, reducing the Aβ load in the brain by 40% while slowing the rate of tau spread. In addition, triple therapies targeting Aβ clearance, tau phosphorylation inhibition, and neuroinflammatory regulation (such as aducanumab + semorinemab + anti-IL-6 antibodies) are being explored, with the preliminary data showing a delay in cognitive decline of approximately 30% [189]. Despite promising prospects, the risk of overlapping toxicity of multitarget drugs and the long-term safety of delivery systems need to be rigorously evaluated.

### 5.3. Gene Therapy

Gene therapy has the potential to modify the genetic risk at the root of AD, especially for familial AD (FAD) and APOEε4 carriers. Delivery of the APOE epsilon 2 gene via adeno-associated virus (AAV) vectors reduces Aβ plaque deposition and improves synaptic function in transgenic mice expressing APOE epsilon 4, and the mechanism may be related to competitive inhibition of APOE epsilon lipoprotein metabolic toxicity [190]. On the other hand, CRISPR/Cas9 technology has been used to edit disease-causing genes precisely, such as by targeting the β-secretase cleavage site of the APP gene in AD models, reducing Aβ production by 80% [191]. However, the challenges of gene therapy are the delivery efficiency (especially the efficient transfection of neurons) and long-term safety, and the potential effects of APOE epsilon 4 regulation on the cardiovascular system still need to be carefully evaluated. In the future, the accuracy of targeted delivery systems based on single-cell sequencing and in situ gene editing technologies is expected to improve.

### 5.4. Neuroprotective Agents

Neuroprotective strategies, with core targets including mitochondrial function repair, oxidative stress inhibition, and the promotion of synaptic regeneration, are designed to delay neuronal degeneration. In a phase II trial (NCT03514875), MitoQ, a mitochondrion-targeted antioxidant, resulted in a 35% reduction in the level of 8-OHdG, a marker of oxidative damage, in the cerebrospinal fluid of patients with AD, along with a 2.5-point improvement in the MMSE score. These findings suggest that MitoQ can maintain neuronal survival by reducing mitochondrial free radical damage. Another class of neuroprotectants with synergistic anti-inflammatory and antioxidant effects includes the curcumin derivative CNB-001, which inhibits microglial overactivation by activating the Nrf2 pathway, reducing the rate of hippocampal atrophy by 20% in patients with mild AD in phase II trials [192]. In addition, an AAV vector-based brain-derived neurotrophic factor (BDNF) delivery system successfully promoted synaptic regeneration in animal models, with its initial safety confirmed in a phase I trial (NCT05040217) [193]. However, the efficacy of neuroprotectants depends on early intervention and needs to address BBB penetration and long-term drug tolerance. In the future, combination protector–disease-modifying therapy regimens combined with biomarker dynamic monitoring may become mainstream.

### 5.5. Multidimensional Intervention Strategies of Marine-Derived Therapeutics

Marine-originated pharmaceuticals, capitalizing on their distinct array of bioactive compounds and structural complexity, exhibit remarkable prospects in advancing polypharmacological approaches for Alzheimer’s disease [194]. Bioactive agents derived from marine species, including sulfated oligosaccharides from brown algae, cyclopeptides from sponges, and halogenated alkaloids from mollusks, possess characteristic molecular modifications that coordinate regulation of AD pathogenesis via cross-talk mechanisms spanning the “gut microbiota-immune-neurological” axis [195]. The regulatory endorsement of GV-971, a sulfated oligosaccharide isolated from brown algae, corroborates the clinical viability of multi-target therapeutic paradigms in AD management [174]. Notably, the marine-derived peptide tasiamide B exemplifies dual-pathway efficacy by suppressing BACE1-mediated Aβ biosynthesis while augmenting microglia-mediated Aβ clearance, thereby reinforcing the capacity of marine-based therapeutics to engage AD’s multifaceted pathological cascades [196]. Collectively, these advancements highlight the intrinsic structural versatility and polypharmacological properties of marine natural products, proposing innovative frameworks to transcend the constraints of single-target drug design. This evidence positions marine-derived agents as promising candidates for next-generation combinatorial therapies targeting AD’s core pathological hallmarks, including dysregulated neuroinflammatory responses, proteostatic dysfunction, and synaptic impairment.

## 6. Conclusions

This review describes the progress of clinical trials and the development of drugs targeting Aβ, tau, the cholinergic system, neuroinflammation, and glutamate for the treatment of AD. AD drug development is shifting from symptomatic treatment to etiological intervention, with Aβ- and tau-targeting drugs, immunotherapy, and multidisciplinary joint strategies as the future focus.

## Figures and Tables

**Table 1:** Differences between primary and secondary Alzheimer’s disease.

Dimension	Primary Alzheimer’s Disease	Secondary Alzheimer’s Disease	References
Incidence/Prevalence	Very common; accounts for 60–80% of all dementia cases	Relatively rare; represents a small proportion of dementia cases	[13]
Gender Ratio	More common in females (approx. 2× higher risk than males); possibly linked to hormonal differences	Varies depending on the underlying cause	[14]
Typical Age of Onset	Usually after age 65 (late-onset AD); early-onset AD (ages 40–65) is less common	Can occur at any age, depending on the underlying condition	[13]
Pathogenesis	Progressive accumulation of Aβ plaques, hyperphosphorylated tau, neurofibrillary tangles, and neuronal degeneration	Highly variable depending on the cause: trauma, tumors, toxins, infections, and metabolic imbalances	[15,16]
Genetic Factors	Significant role, especially in early-onset AD; APOE ε4 allele is a known risk factor	Genetics are usually not involved, unless the primary disease itself has a hereditary basis	[17,18]

## Data Availability

No primary data were generated as part of this work.

## Abbreviations

The following abbreviations are used in this manuscript:

AAV	adeno-associated virus
ACh	acetylcholine
AChE	anticholinesterase
ACTION	ACTivities of daily living and cognitION
AD	Alzheimer’s disease
ADAS-Cog	Alzheimer’s Disease Assessment Scale–Cognitive Subscale
ADL	activities of daily living scale
AEs	adverse events
AES	Apathy Evaluation Scale
APP	amyloid precursor protein
ARIA-E	amyloid-related image abnormality of edema or effusion
ARIA-H	amyloid-related imaging abnormalities-hemosiderin
ARIAs	amyloid-related imaging abnormalities
Aβ	amyloid-beta
Bace1	β-secretase
BBB	Blood-brain barrier
BDNF	brain-derived neurotrophic factor
CDR-SB	Clinical Dementia Rating Scale-Sum of Boxes
CGIC	Clinical General Impression Change
ChAT	activity of choline acetyltransferase
CIBI	Clinician Interview-Based Impression
CNS	central nervous system
CSF	cerebrospinal fluid
DAT	Dementia of Alzheimer Type
FCCA	Final Comprehensive Consensus Assessment
FDA	the U.S. Food and Drug Administration
GLP-1RA	glucagon-like peptide-1 receptor agonist
IADL	Instrumental Activities of Daily Living
LMT	leuco-methylthioninium
MCI	mild cognitive impairment
mGLuR	metabotropic glutamate
MMSE	Mini-Mental State Examination
MPAC	metal-protein attenuating compound
MPH	Methylphenidate
MRI	magnetic resonance imaging
NE	norepinephrine
NET	noradrenaline transporter
NIH	National Institutes of Health
NMDA	N-methyl-D-aspartic acid receptor
PDS	Progressive Deterioration Scale
ROS	reactive oxygen species
SIB	Severe Impairment Battery
